# Assessment of displacement ventilation systems in airborne infection risk in hospital rooms

**DOI:** 10.1371/journal.pone.0211390

**Published:** 2019-01-30

**Authors:** José Manuel Villafruela, Inés Olmedo, Félix A. Berlanga, Manuel Ruiz de Adana

**Affiliations:** 1 ITAP, Department of Energy and Fluid Mechanics, University of Valladolid, Valladolid, Spain; 2 Department of Physical Chemistry and Applied Thermodynamics, University of Cordoba, Córdoba, Spain; Instituto Nacional de Astrofisica Optica y Electronica, MEXICO

## Abstract

Efficient ventilation in hospital airborne isolation rooms is important vis-à-vis decreasing the risk of cross infection and reducing energy consumption. This paper analyses the suitability of using a displacement ventilation strategy in airborne infection isolation rooms, focusing on health care worker exposure to pathogens exhaled by infected patients. The analysis is mainly based on numerical simulation results obtained with the support of a 3-D transient numerical model validated using experimental data. A thermal breathing manikin lying on a bed represents the source patient and another thermal breathing manikin represents the exposed individual standing beside the bed and facing the patient. A radiant wall represents an external wall exposed to solar radiation. The air change efficiency index and contaminant removal effectiveness indices and inhalation by the health care worker of contaminants exhaled by the patient are considered in a typical airborne infection isolation room set up with three air renewal rates (6 h^-1^, 9 h^-1^ and 12 h^-1^), two exhaust opening positions and two health care worker positions. Results show that the radiant wall significantly affects the air flow pattern and contaminant dispersion. The lockup phenomenon occurs at the inhalation height of the standing manikin. Displacement ventilation renews the air of the airborne isolation room and eliminates the exhaled pollutants efficiently, but is at a disadvantage compared to other ventilation strategies when the risk of exposure is taken into account.

## Introduction

Hospital facilities are places with a high risk of cross infection between their occupants. Studies in European hospitals [1] indicate that nosocomial infections contribute significantly to morbidity and mortality rates and that many of these infections are transmitted by airborne pathogens [2]. Everyday pulmonary activities, like breathing [3], coughing [4,5], sneezing [6], talking [3], are sources of bio-aerosols [7] that may be laden with the pathogens responsible for infectious disease transmission. Once the bio-aerosols leave the infected person their fate depends on multiple and complex factors [7–10]. One of the most important factors is undoubtedly the airflow pattern, both in the room as a whole as well as in the microenvironment around the source patient and the vulnerable individual.

### Airborne infection isolation room

If the appropriate measures are not taken, the bio-aerosols emitted by patients hospitalized with an airborne disease may be dispersed uncontrollably around the airborne infection isolation room (AIIR) or the rest of the hospital [11]. Different methods and technologies are available to provide adequate protection to people who pass through a hospital [12]. One of the recommended measures is to maintain a negative pressure with respect to the surrounding area so that air flows into the room and not in the opposite direction when doors are open. Unfortunately, negative pressure briefly disappears during door operation and air leakage is virtually inevitable [13]. Many guidelines and regulations [14–18] related to airborne isolation rooms (AIIR) advise or require that access to the room should be through an anteroom in order to minimize escape of contaminated air [19,20]. Yet neither the negative pressure nor the anteroom prevents the risk for the person entering the AIIR. Only personal self-protection measures and a suitable ventilation strategy reduce the possibility of contagion [21].

With regard to ventilation, one common recommendation is to use high renewal rates to dilute and remove pathogens [22]. However, this does not prevent the appearance of stagnant zones and short-circuiting, resulting in “clean” and “polluted” areas of exhaled pathogens with the subsequent risk of high cross-infection rates. Several studies indicate that the design of a ventilation system and the resulting airflow patterns play a more important role than just air renewal rates alone [23,24]. Airflow patterns generated by ventilation systems can be controlled, and recent research has focused on providing good air distribution rather than on maintaining high rates of air renewal as a strategy to reduce the risk of airborne contagion [25–30].

### Displacement ventilation

Various ventilation strategies such as mixing ventilation (MV) and displacement ventilation (DV) offer different possibilities to protect people from airborne cross infection [10,31]. MV is the most widely applied strategy in hospital patient rooms. However, in recent years DV has emerged as an alternative. Some studies have shown that DV is more energy efficient [32]. Standardisation associations have developed DV guidelines and recommendations for designers.

DV systems were initially developed to remove thermal loads in industrial warehouses due to their ability to concentrate heat and pollutants above the occupied zone. DV systems are characterized by thermal and mass stratification such that they cannot be modelled with the fully mixed room air approach [33]. In DV, cool air is supplied into the lower part of the room using low impulse diffusers. This slow moving fresh air fills the room from below, is heated and rises to the ceiling, where the exhaust is located. There must be heat sources for DV to work. As breathing is also a heat and pollutant source, contaminants might be transported directly to the upper part of the room. DV offers the possibility of working with two zones, a low zone with clean air, and an upper zone with pollutants. Some authors report that it is possible to design DV hospital patient rooms that have low human exposure to bio-aerosols that containing pathogens [32,34], although in certain situations high exposure may also exist in rooms with DV [35–37].

### Objective and methodology

The aim of this work is to evaluate the suitability of applying the DV strategy in AIIRs. The analysis is mainly based on numerical simulation results obtained with the support of computational fluid dynamics (CFD). The interaction between the different air flows -breathing flows [38], convective flows around human bodies [39], thermal plumes above heat sources, rising boundary layer flow at the warm wall together with large-scale air movements due to room air flow instabilities- is so complex that it is difficult to approach the problem directly. Initially, dispersion of contaminants exhaled by a single person standing in an indoor environment was studied [40]. Later, a second person facing the first was added to analyse the interaction between the respiration flows of both people [41]. These two previous studies have enabled an adequate procedure to be established for analysing the role of ventilation in the risk of cross-infection between patient and susceptible health care worker caused by the airborne pathogens exhaled during breathing in an AIIR with DV. Using the validated model, twelve different numerical tests are carried out to analyse how air renewal rates, the position of the health care worker, and the position of air exhaust openings affect the risk of cross infection.

### Test room and experimental setup

**Fig 1:**
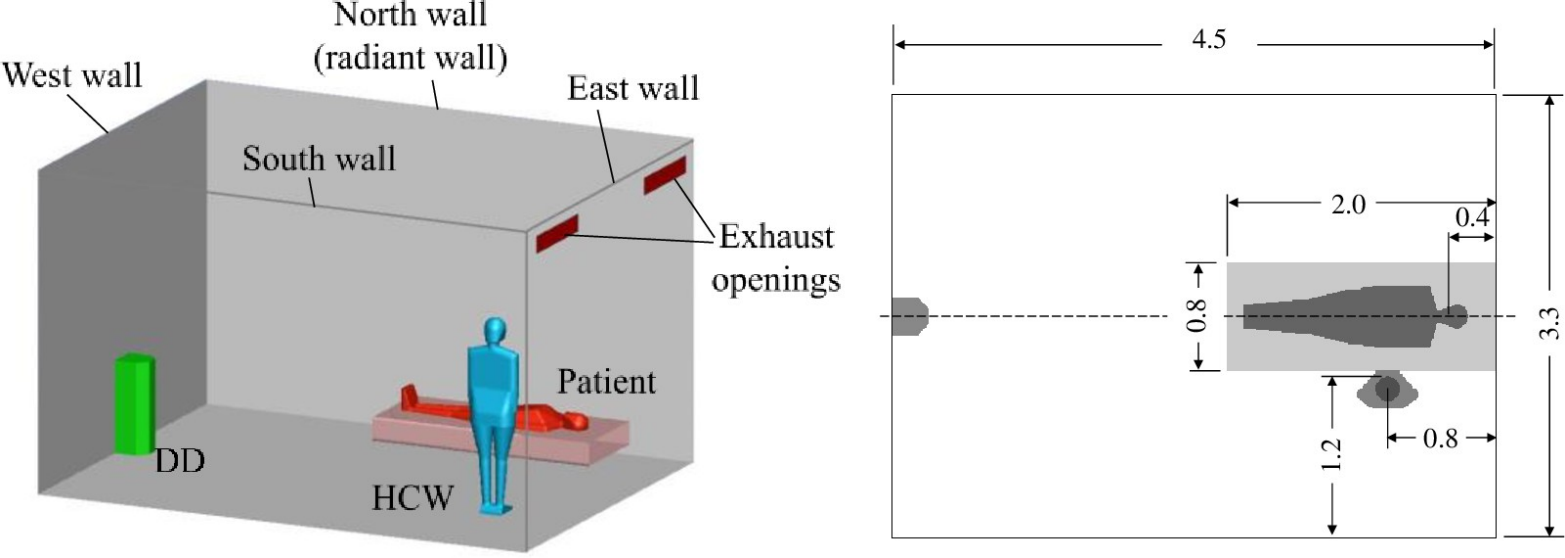
Experimental setup. Numerical setup for EN cases (Table 1).

The experimental study of a patient (P) lying on a hospital bed and a health care worker (HCW) standing close to the bed in a typical AIIR room (Fig 1) [14,15,42,43] was carried out in a test room at Cordoba University, 4.5 m (long), 3.3 m (wide), and 2.8 m (high). The two thermal breathing manikins have the same geometry. The total sensible heat emitted for each manikin corresponds to a metabolic rate of 1 met for the HCW and 0.7 met for the patient, 80 W and 70 W, respectively. There is an external heat gain of 500 W in the 4.5 m wall opposite the HCW, which represents an external wall exposed to solar radiation. The remaining walls as well as the floor and ceiling are adiabatic as the chamber is inside a lab at the same temperature.

A displacement flow diffuser (QLV-180-200-800, Trox, Germany) are used as supply air unit for the hospital room, and two exhaust openings were located on the opposite wall, just below the ceiling. The ventilation system was set at three different air change rates of 12, 9 and 6 ACH, supplying air at 21.8°C, 20.6°C and 18.2°C, respectively to maintain the same mean room temperature. Part of the effective area of the displacement diffuser is partially covered during the 9 ACH and 6 ACH tests in order to maintain the same supply velocity. Information about breathing manikins, measuring instruments and others details of these experiments can be found in [44].

**Table 1:** Conditions of the CFD test performed.

Simulation nomenclature	Exhaust openings wall	Radiant wall	ACH	Supply air flow (m^3^/h)	Supply air temperature (°C)	Simulation time (min)
EN_12*	East	North	12	500	21.8	10
ES_12	East	South				
WN_12	West	North				
WS_12	West	South				
EN_09*	East	North	9	375	20.6	15
ES_09	East	South				
WN_09	West	North				
WS_09	West	South				
EN_06*	East	North	6	250	18.2	20
ES_06	East	South				
WN_06	West	North				
WS_06	West	South				

(*) Experimental test

## Numerical simulation

### Indices for quantifying the ventilation and infection risk

The ventilation system of a room can pursue different aims: thermal comfort, air renewal, elimination of gaseous or suspended contaminants, avoiding risk of infection, etc. Depending on the activities carried out in the room, one aim or another will prevail. Specific indices exist to quantify the extent to which each aim is achieved.

The most basic index is *air changes per hour* (ACH). To calculate ACH, only the air volume of the room and the air flow rate need to be known. This index is commonly used in guidelines and recommendations.

The *air change efficiency index* (*ε*_*a*_) is defined as the ratio between the minimum and the actual mean replacement times and can be calculated from the expression:

$$\varepsilon_{a}=\frac{\tau_{n}}{2\;\bar{\tau }}$$ (1)

where *τ*_*n*_ = *V*/*Q*, *V* is the room volume and *Q* is the flow rate of fresh air, i.e. *τ*_*n*_ is the inverse of the number of air changes per second, and $\bar{\tau }$ is the average age of the air in the room. Air change efficiency only depends on the overall air flow pattern in the room, and takes values between 0 and 1.

If, in addition to knowing the ACH and the air flow pattern, the characteristics of the contaminant and the point of emission are also known, then the *contaminant removal effectiveness index* (*ε*_*c*_) can be used. This index can take any positive value. Assuming that the air supplied to the room is contaminant free and that the flow is steady, *ε*_*c*_ is calculated by dividing the concentration of pollutant in the exhaust air *c*_*e*_ by the average concentration in room $\bar{c}$:

$$\varepsilon_{c}=\frac{c_{e}}{\bar{c}}$$ (2)

Finally, if the area to be protected is known -the surface of a printed circuit during manufacture, the instrument table during a surgical operation or the lungs of a person sharing a room with another infected person- the intake fraction (*IF*) index may be used. This index is the flow rate of contaminant that crosses the surface to be protected divided by the flow rate of contaminant that enters or is generated inside the room (Bennett et al., 2002). In order to assess the risk of cross-infection, the intake fraction is defined as the proportion of the cumulative mass of contaminant inhaled by the HCW to the mass of contaminant emitted in the patient's exhalation during the same period of time.

$$IF=\frac{\int Q_{HCW}Y_{HCW}dt}{\int Q_{P}Y_{P}dt}$$ (3)

Where *Q*_*HCW*_, and *Q*_*P*_ are the instantaneous breathing flow rate of HCW and P, respectively, *Y*_*HCW*_ is the instantaneous mass fraction of N_2_O in the HCW inhalation air, and *Y*_*P*_ is the instantaneous mass fraction of N_2_O in the patient’s exhalation air.

### Governing equations

Airborne cross infection between occupants is unsteady, non-isothermal and is a three-dimensional problem involving two species: air and contaminant. As modelling tool, CFD has been applied to simulate the unsteady airflow using the URANS method together with the RNG k–e turbulence model equations, mean age of air equation, the N_2_O mass fraction equation and includes the effect of thermal radiation using commercial software Ansys Fluent.

The local mean age of air *τ* in the whole fluid field is calculated solving the following conservation equation:

$$\frac{\partial \tau }{\partial t}+\nabla (\tau \vec{v}-D_{a}\nabla \tau )=1$$ (4)

where $\vec{v}$ is the air velocity and *D*_*a*_ is the mass diffusion coefficient [45]. A subroutine solving Eq (4) numerically is written, and the subroutine is built into the CFD-program. Once the average age of the air in the whole room is calculated, it is possible to directly evaluate *ε*_*a*_ according to Eq (1). $\bar{\tau }$ is the average of *τ* in the whole room and *τ*_*n*_ can be calculated as the inverse of the number of air changes per second or as the average of *τ* in the air extractions. The two values coincide.

The CFD-program models the mixing and transport of two chemical species, air and N_2_O, by solving equations describing convection and diffusion for each component species without reactions. *ε*_*c*_ and *IF* can be calculated directly from their definitions. Since the model is transient, the time evolution of *ε*_*c*_ and *IF* can be calculated.

Radiation is introduced into the CFD model using the surface-to-surface radiation model [46]. The importance of thermal radiation in airflow with DV was examined experimentally in [47]. The RNG *k*–ε model that takes into account the low Reynolds-number effects in conjunction with enhanced wall treatment that combines a two-layer model with enhanced wall functions are used in these simulations. Pressure-velocity coupling was resolved using the PISO scheme. A second-order implicit transient formulation is chosen which is unconditionally stable with respect to time-step size. A second-order upwind discretization scheme is used for all equations [46].

In transient simulations (also in experiments) an error might occur during start-up and when letting simulations run sufficient time to achieve characteristic large eddy turnover time [48]. The initial conditions for non-steady computations are obtained for a steady simulation. The first 30 minutes of the transient simulation are discarded. Large eddy turnover time is a characteristic timescale for the domain *l*_0_/*v*_0_ where *l*_0_ is the largest scale of the room and *v*_0_ is the characteristic velocity. An estimation of *v*_0_ can be made by dividing the ventilation flow rate by a half section of the test chamber, which gives a large eddy turnover time of five minutes for 6 ACH. A large eddy turnover time is inversely proportional to ACH if the remaining parameters remain unchanged. In order to obtain a suitable temporal average, total times of 20, 15 and 10 minutes are simulated for 6, 9 and 12 ACH, respectively (Table 1). To capture the effects of the smaller time scales related to the breathing process, a time step of 0.02 s is selected.

### Computational domain

The domain of the computational model mimics in detail the experimental geometry of the life-size hospital isolation room. Most of the domain is built with a hexahedron mesh. A tetrahedral mesh has been employed near the diffuser and near to the manikin’s surface due to their geometry complexity. Mesh refinement was performed around the manikins, the exhausts, the walls, and the displacement diffuser since high velocity and temperature gradients are expected. The high concentration, velocity and temperature gradients require a very fine local grid system at the manikins’ faces and in the exhalation zones [49]. The shape of the manikins for the numerical simulations reproduces a thermal breathing manikin used by others authors [50,51]. Detailed information about the manikins’ shape and mesh can be found in [41]. A sensitivity study was carried out with successive refinements of the exhalation zones and around the displacement diffuser. A final mesh of nearly one and a half million cells is used.

### Boundary conditions

The CFD model of a patient and a HCW in AIIR faithfully reproduces the experimental conditions [44]. The lying manikin (also known as the patient or source manikin, SM) exhales through the mouth and inhales through the nose. The standing manikin (also known as the HCW or target manikin, TM) exhales and inhales through the nose. Breathing functions are a very important point in these simulations [52]. The two manikins breathe following a sinusoidal function. For the patient manikin, the tidal volume is 0.57 litres and the breathing frequency is 20 breaths/minute. For the HCW manikin, the tidal volume is 0.66 litres and the breathing frequency is 15 breaths/minute. The patient manikin thus performs four full breaths in a 12-second period and the HCW manikin three full breaths during the same period. Velocity is normal and uniform in the HCW’s nostrils and in the patient’s mouth. The temperature of expired air in the two manikins is 34°C. The mass fraction of N_2_O in the exhaled air of the patient manikin is *Y*_*P*_ = 0.027.

The boundary condition for the displacement diffuser is a uniform velocity of 0.926 m/s, which is normal for vertical diffuser surfaces. For the 12 ACH simulations, the entire front area of the displacement diffuser is used as an inlet. The upper quarter and upper half of the displacement diffuser front area are considered as walls for the 9 and 6 ACH simulations, respectively, in order to maintain the same inlet velocity [53]. The effective area of the displacement diffuser is taken into account adding the corresponding momentum/volume source in a sub-domain in front of the diffuser [54].

The air leaves the room through two exhaust openings located in the same wall as the displacement diffuser (west) or in the opposite wall (east). A pressure-outlet boundary condition is imposed in the exhaust openings.

In order to maintain the same mean indoor temperature for all tests, the air is supplied at a temperature of 21.8°C, 20.6°C, and 18.2°C for 12, 9 and 6 ACH, respectively. The ceiling, floor and all the walls except one 4.5 m × 2.8 m wall (north or south wall) are considered adiabatic. In this non-adiabatic wall a heat flow of 39.7 W/m^2^ is imposed as the boundary condition. This represents a glazed wall with a transmission coefficient of 4 Wm^-2^K^-1^ with 10°C temperature difference between indoor and outdoor air. The lying and standing manikins have a thermal load of 70 W and 80 W, respectively.

## Validation of numerical results with experimental data

In rooms with displacement ventilation, thermal stratification is generated. The temperature of the exhaled air is higher than the temperature of the ambient air. The effects of buoyancy will play an important role in both the evolution of exhaled air and the dispersion of the associated pathogens. It is therefore crucial to correctly predict the room’s temperature field. This numerical model was previously used to study the dispersion of exhaled contaminants by the mouth of a person standing in a room with DV. The numerical results were compared to data from experiments performed in a full-scale laboratory at the University of Aalborg [55]. It was found that the numerical model was able to accurately reproduce both the thermal stratification in the room and the deflection of the exhaled air jet. Details on the validation of the temperature field are shown in [40].

Exhaled pathogens do not disperse in the same way when expired through the nose or mouth. How the vulnerable individual breathes also affects the microenvironment near the face and, therefore, the risk of inhaling pathogens. This CFD model was used in a previous study to analyse how the way of breathing influences the risk of cross-infection between two people standing facing each other at different distances in a room with DV. The results of the CFD simulations were compared to experimental data obtained in the Alborg laboratory with two manikins at different distances and exhaling through the mouth and inhaling through the nose. Details of this new validation are shown in [41].

**Fig 2:**
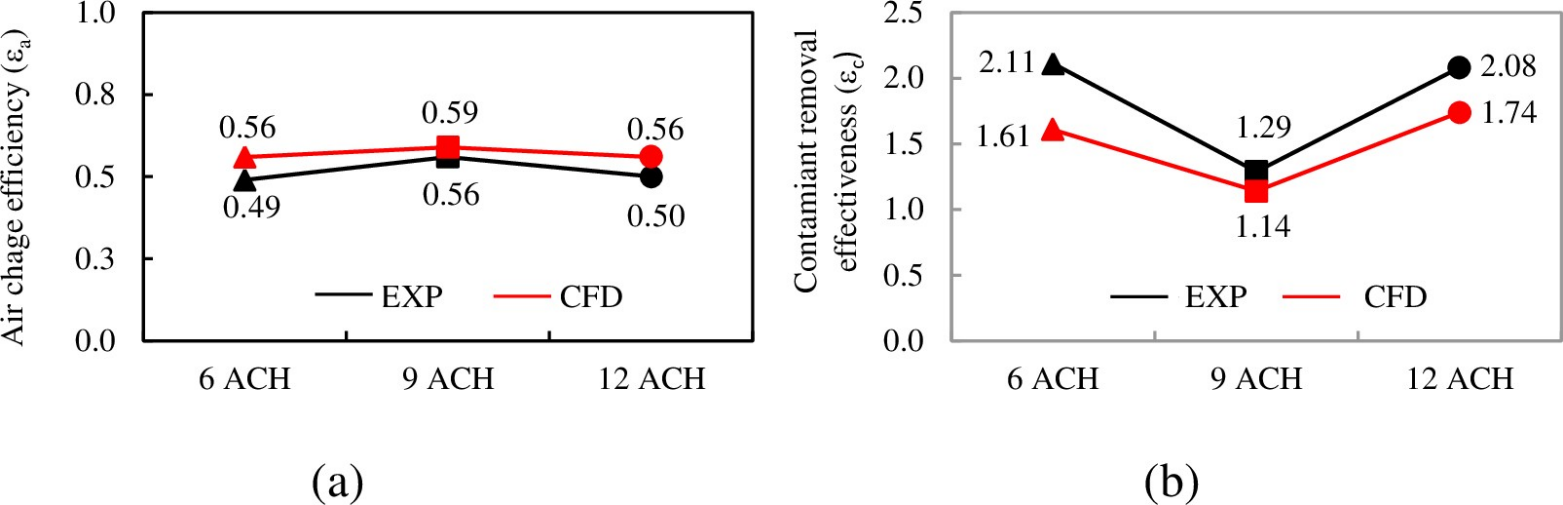
Experimental and numerical air change efficiency index. a) Air change efficiency. b) Contaminant removal effectiveness. EN cases.

The numerical model is now validated again with the experimental results obtained in a real-scale laboratory at the University of Córdoba [44]. Comparing the results of two global ventilation efficiency indices, the air change efficiency *ε*_*a*_ and the contaminant removal effectiveness *ε*_*c*_ clearly evidences how the numerical model is able to capture the experimental tendencies and to reproduce the values to a reasonable degree of concurrence (Fig 2). The experimental values of contaminant removal effectiveness are slightly higher than the numerical values. The possibility that these differences are due to difficulties that emerge when measuring these indexes using photoacoustic spectroscopy cannot be ruled out.

## Results and discussion

### Temporal evolution of contaminant inhaled

**Fig 3:**
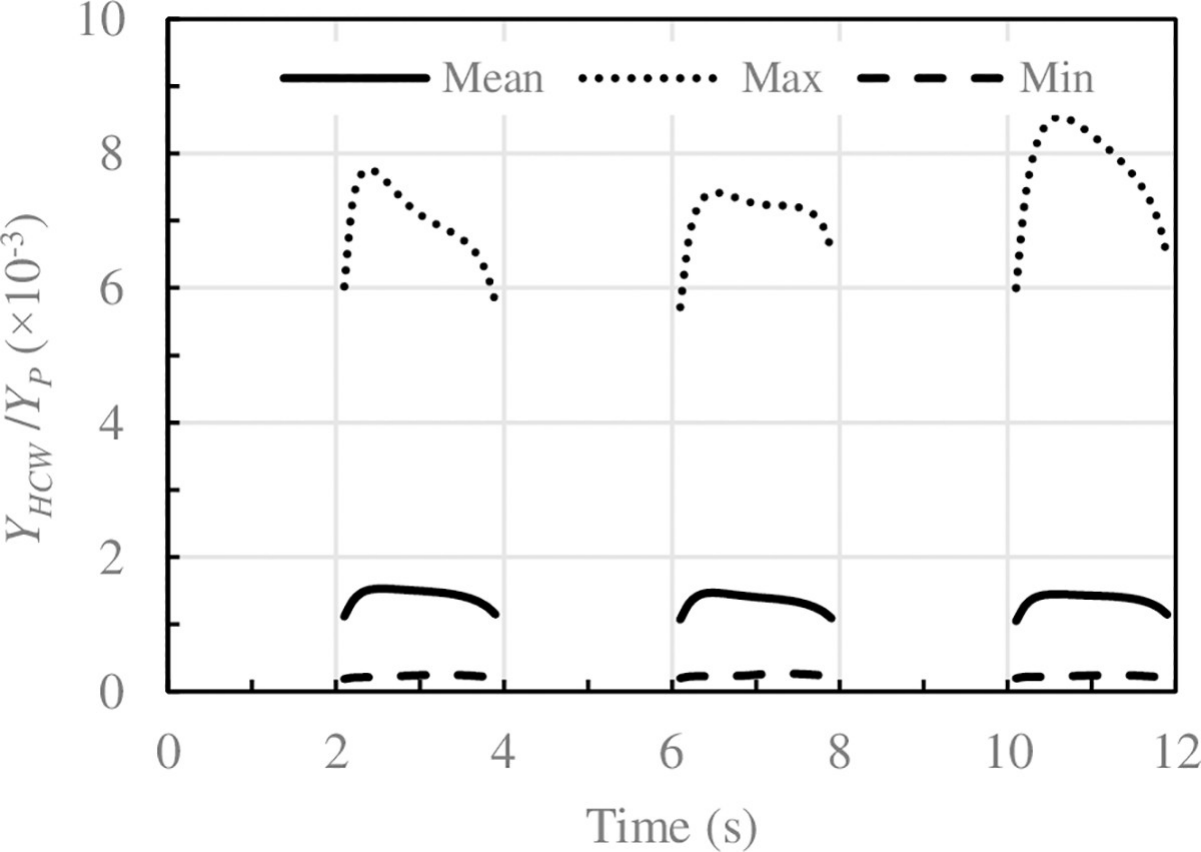
Concentration of N_2_O inhaled by the HCW for the case of EN_09. Solid lines: phase-average values, dotted lines: maximum value, dashed lines: minimum value corresponding to 75 cycles of 12s.

The exhalation, dispersion and inhalation of contaminants are transient phenomenon. Numerical data provide a detailed temporal evolution of the amount of contaminant inhaled by the HCW. A breathing cycle (inhalation and exhalation) lasts three seconds for the P and four seconds for the HCW. Each 12 seconds both start at the same time, although the two breaths are out of phase throughout the cycle. This progressive phase displacement might cause the amount of contaminant inhaled by the HCW in each of the three cycle inhalations to differ. The phase-averaged method was used in Fig 3 to show the concentration of N_2_O inhaled by the HCW in 12-second cycles. It is worth noticing that the average amount of contaminant inhaled in the three inhalations is the same. This is a clear indication that the interaction between the breaths of the two manikins is very weak due to the great distance between the breathing zones of the two manikins [41]. Between the mouth of the patient and the nose of the HCW there is a distance of 0.94 m in a straight line. The nose of the HCW is 0.53 m away from the patient's exhalation axis.

It should also be mentioned that the amount of pollutant inhaled in each 12-second cycle changes significantly. In other words, cyclical dispersion is very noticeable, especially for low ACH. This result shows that the dispersion of exhaled contaminants and their subsequent inhalation are transient phenomena due to the transient nature of the airflow pattern in the area between P and HCW. The overall airflow in the room also exhibits a transient behaviour with room airflow frequencies lower than the manikins’ breathing frequencies. These results are in line with previous works [41,56].

### Airflow pattern. Air change efficiency

In a perfect mixing ventilation (PMV) flow, air composition is equal throughout the whole room and no contaminant concentration gradients are present. PMV implies infinitely rapid diffusion and perfect displacement ventilation PDV implies absolute absence of diffusion [45]. PMV and PDV are idealized theoretical flow patterns that never occur in practice but which are, nevertheless, useful concepts for comparative purposes. In a perfect mixing ventilation flow *ε*_*a*_ = 0.5. In a perfect displacement ventilation flow *ε*_*a*_ = 1.

**Fig 4:**
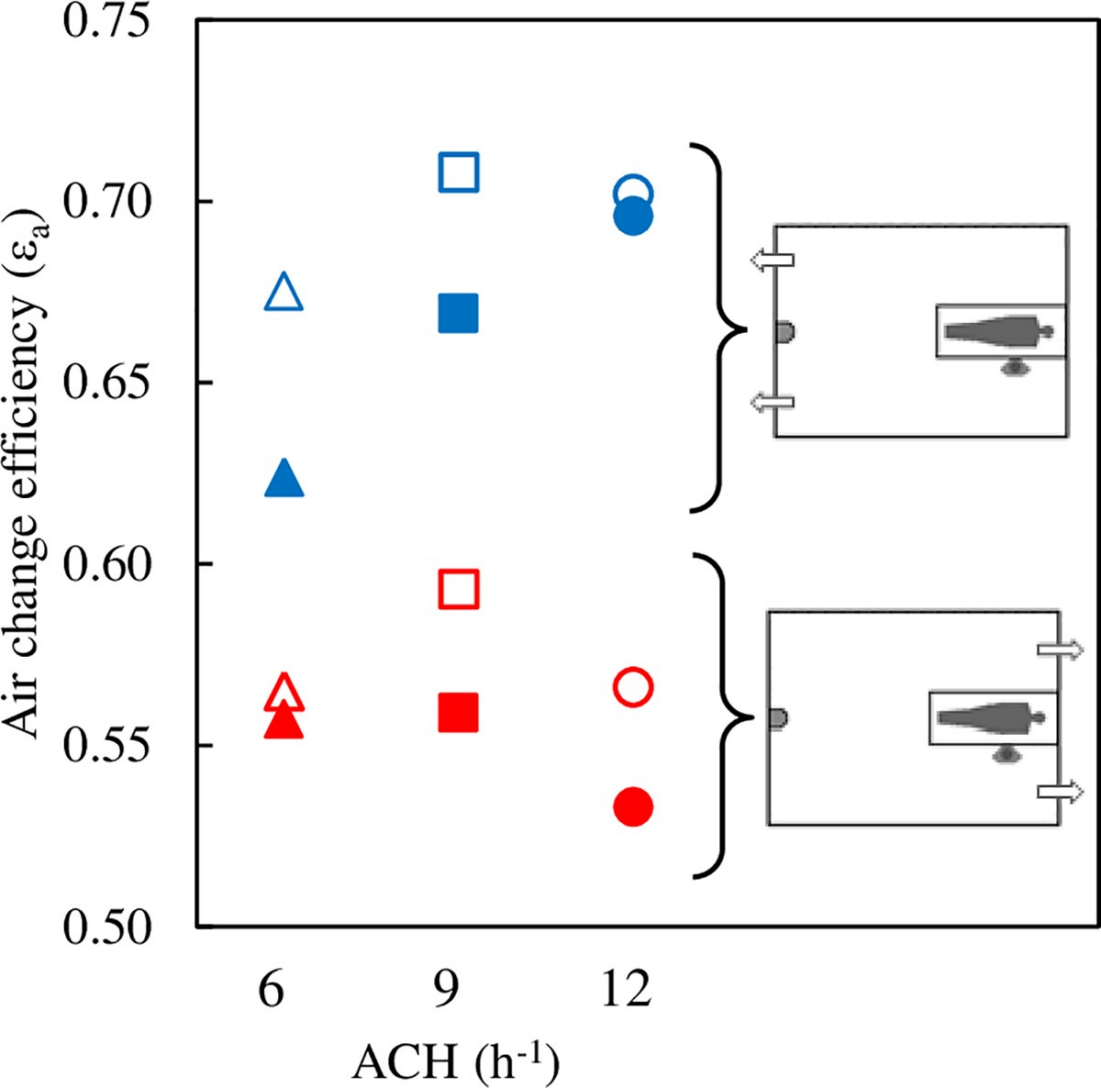
Air change efficiency index. Blue symbols: exhaust openings on the wall of the DD. Red symbols: exhaust openings on the wall of the bed. Full symbols: radiant wall in front of the HCW. Empty symbols: radiant wall behind the HCW.

As expected, in all the cases analysed, 0.5<*ε*_*a*_<1.0, when using the DV strategy. There is a clear correlation between the ventilation efficiency and the position of the air exhaust openings (Fig 4). With the exhaust openings in front of the displacement diffuser, in the wall of the bed, average ventilation efficiency is *ε*_*a*_ = 0.56. With the exhaust openings in the wall opposite the bed the average efficiency increases significantly, *ε*_*a*_ = 0.69. The air flow pattern in the room is closer to that of PDV. This behaviour is in line with other authors’ observations.

The air change efficiency index is also correlated with the position of the radiant wall. In all cases, when the radiant wall is behind the HCW (empty symbols) air change efficiency is greater than when it is opposite the HCW (full symbols), probably because thermal loads are more balanced compared to the room’s plane of symmetry. No clear correlation can be established between air change efficiency and ACH.

### Dispersion of the contaminant. Contaminant removal effectiveness

**Fig 5:**
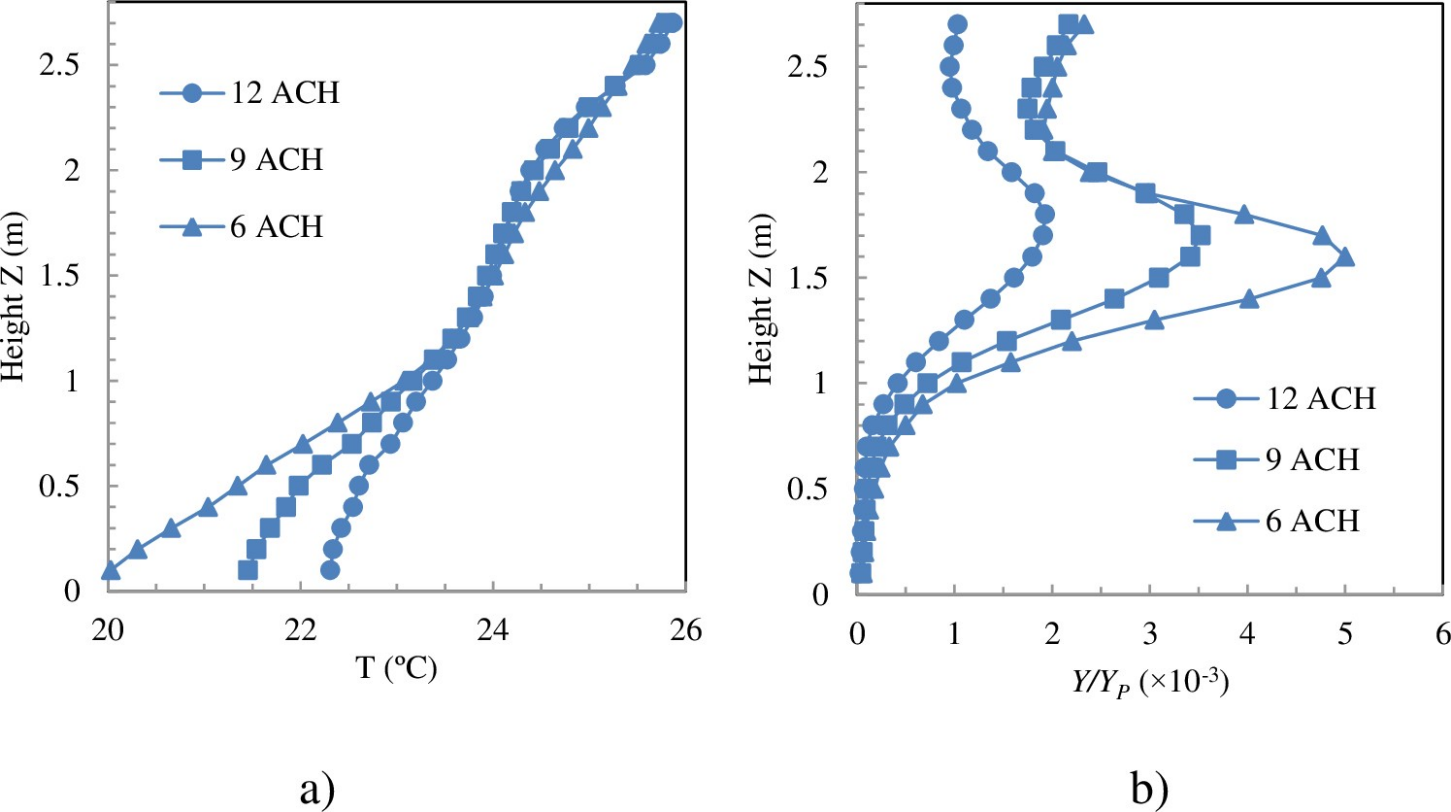
Temperature and percentage of pollutant averaged over time and over vertical planes. Exhaust openings on the bed and radiant wall in front of the HCW (Cases WN).

DV provides vertical thermal stratification that determines contaminant dispersion in the room. In order to maintain the same mean temperature in the room with less ACH, the difference between the air inlet temperature and the average temperature of the room is increased. Up to a height of 1.1 m, the vertical temperature gradient is greater for low ACH (Fig 5A), whereas from 1.1 m to the ceiling the gradients are almost equal for all ACH. This behaviour can also be seen for the other simulated cases. These numerical results are consistent with experimental observations [57,58].

As the contaminant source is also a source of heat, the contaminant will be transported directly to the top of the room [59]. The pollutant is expected to be distributed according to a two-zone model [35], a clean zone in the lower part (*y<y*_*st*_) of the room and an unclean zone in the upper part (*y>y*_*st*_). The mean concentration of N_2_O in horizontal planes is shown in Fig 5B. Below the patient exhalation height, z = 0.78 m, N_2_O concentration is practically negligible. The general airflow pattern in the room, the momentum of the patient's exhalation jet and the convective effects of the exhalation jet and the thermal plume that forms above the patient cause the pollutant to rise. The average N_2_O concentration in horizontal planes increases rapidly, reaching the maximum at a height between 1.6 m and 1.7 m (Fig 5B). The exhaled air is concentrated at the height of the HCW’s head. This behaviour is the so-called lockup phenomenon and has recently been studied numerically [59] and experimentally [60]. The lockup phenomenon was more intense when the ventilation rate decreased, since a low ventilation rate causes a large temperature gradient [59]. The stratification height increased as the ventilation rate increased.

**Fig 6:**
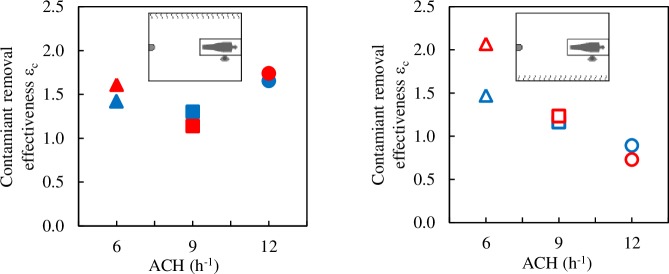
Contaminant removal effectiveness. Blue symbols: exhaust openings on the wall of the DD, red symbols: exhaust openings on the wall of the bed. Full symbols: radiant wall in front of the HCW. Empty symbols: radiant wall behind the HCW.

In a PMV flow, the concentration of contaminant in the room is homogenous, such that the concentration in the exhaust air is the same as the mean concentration in the room, with contaminant removal effectiveness being 1. In all the cases analysed in the present study, contaminant removal effectiveness *ε*_*c*_ is between 0.73 and 2.0 (Fig 6). Only when the radiant wall is behind the HCW and ACH is 12, is *ε*_*c*_ below 1.0. No clear correlation could be established between *ε*_*c*_ or with the position of the radiant wall or the ACH or with the position of the exhaust openings. Moreover, unlike what was observed with *ε*_*a*_, it seems that the exhaust positions barely influence *ε*_*c*_.

**Fig 7:**
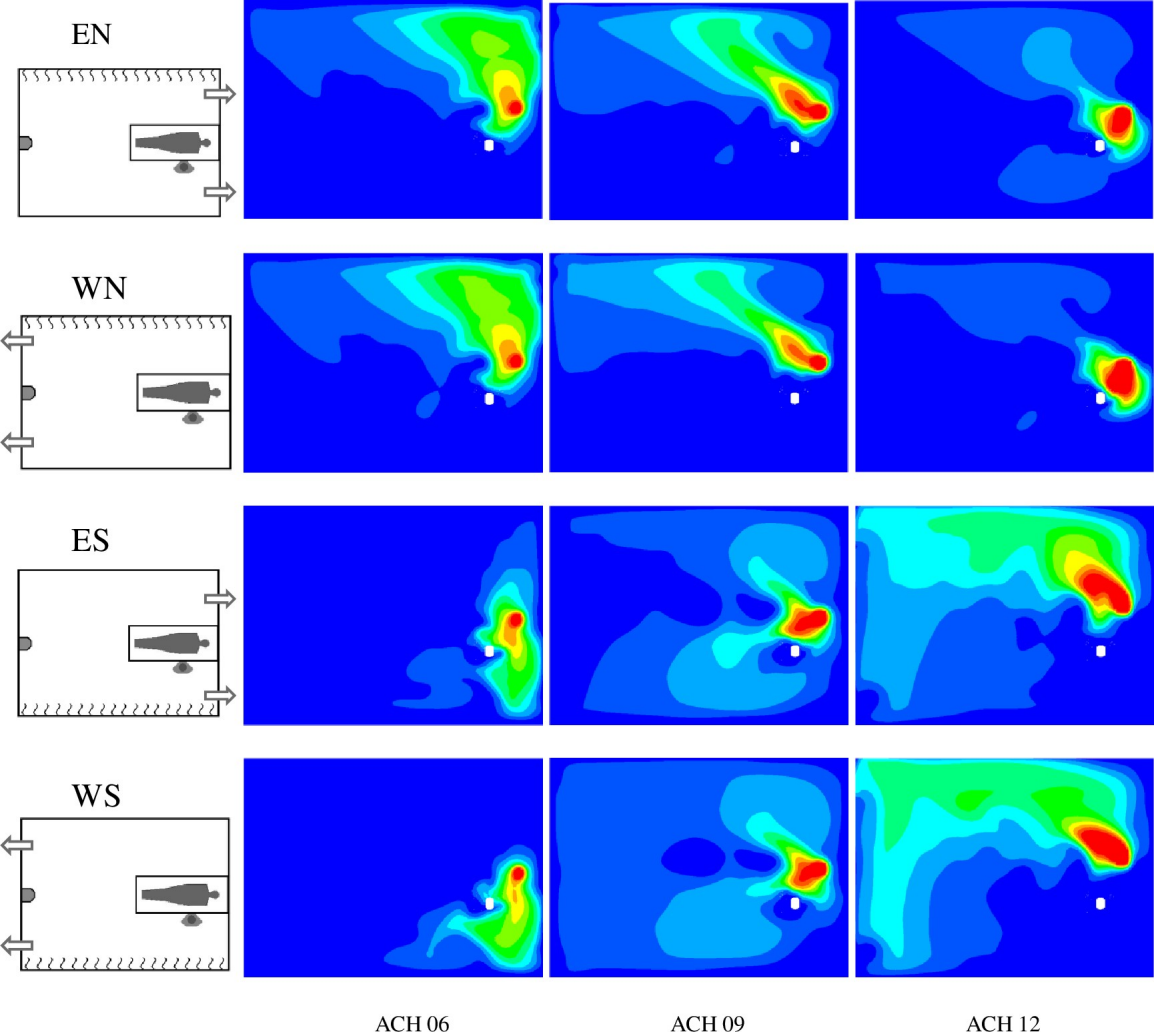
Contours of *Y*/*Y*_*PMV*_ in a z = 1.52 m plane. Dark blue: *Y* < *Y*_*PMV*_ Red: *Y* > 10*Y*_*PMV*_.

In a room with PMV and steady state conditions, the contaminant concentration, *Y*_*PMV*_, would be homogeneous and inversely proportional to ACH:

$$Y_{PMV}=Y_{P}\frac{Q_{P}}{Q_{S}+Q_{P}+Q_{HCW}}\approx Y_{P}\frac{Q_{P}}{Q_{S}}$$ (5)

where *Q*_*P*_ is the patient’s tidal volume by their breathing frequency, *Q*_*HCW*_ is the HCW tidal volume by their breathing frequency, and *Q*_*s*_ is the supply air flow. The *Y*_*PMV*_ values for 6, 9 and 12 ACH are 0.0028 *Y*_*P*_, 0.0018 *Y*_*P*_ and 0.0014 *Y*_*P*_, respectively. Between approximately 1.3 m and 1.9 m in height ˗the HCW inhales at a height of 1.52 m˗ the mean N_2_O concentration in horizontal planes is higher than the corresponding *Y*_*PMV*_ (Fig 5B). However, this does not mean that the HCW inhales air with that mean concentration as can be seen by comparing Fig 3 and Fig 5B. Firstly, pollutant distribution in horizontal planes is not even and, secondly, the convective boundary layer around the HCW enters the air from the lower part, leading to the HCW breathing zone. Fig 7 shows the normalised distribution of N_2_O averaged over time in horizontal planes at the inhalation height.

The distribution of contaminant in z = 1.52 m planes clearly depends on the position of the radiant wall and the ACH number but is barely influenced by the position of the exhaust openings. When the radiant wall is opposite the HCW, the contaminant cloud is deviated towards the radiant wall away from the HCW. This may be due to the convective effects generated by the radiant wall and the horizontal momentum of HCW exhalation. However, when the radiant wall is behind the HCW, the contaminant is distributed more symmetrically with respect to the room’s vertical plane of symmetry. The lack of symmetry in N_2_O distribution at z = 1.52 m affects N_2_O concentration in each of the two extractions. This tendency is less pronounced for the 12 ACH cases.

### Infection risk. Intake fraction

The intake fraction index was obtained as the ratio between the mass of N_2_O inhaled by the HCW during the time simulated and the total mass of N_2_O exhaled by the patient at the same time. In a room with PMV and steady state conditions, the intake fraction index, *IF*_*PMV*_, would be homogeneous and inversely proportional to ACH:

$${IF}_{PMV}=\frac{Y_{PMV}}{Y_{P}}\frac{Q_{HCW}}{Q_{P}}$$ (6)

**Fig 8:**
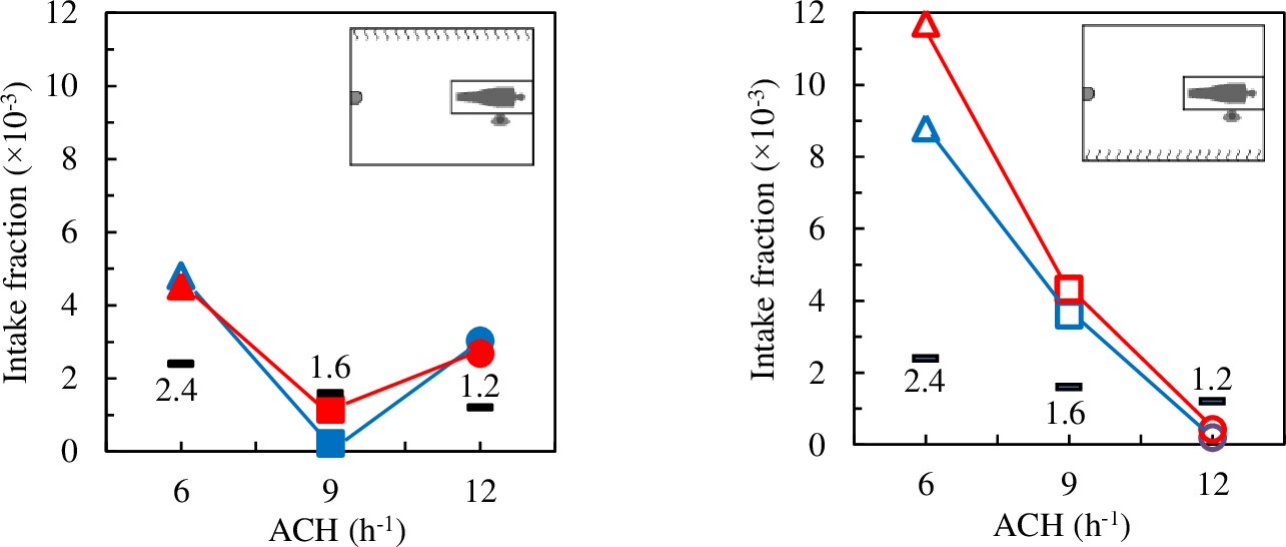
Intake fraction index. Black: *IF*_*PMV*_. Blue symbols: exhaust openings on the wall of the DD, red symbols: exhaust openings on the wall of the bed.

If the breathing rate by tidal volume of both manikins were equal then *IF*_*PMV*_ = *Y*_*PMV*_/*Y*_*P*_. The *Y*_*PMV*_ values for 6, 9 and 12 ACH are 0.0024, 0.016 and 0.0012, respectively. Fig 8 shows the *IF* values for all the DV cases analysed together with the *IF* values that would correspond to PMV. As ACH increases, *IF* values are expected to decrease. However, when the radiant wall is opposite the HCW (Fig 8A) the lowest *IF* values are for 9 ACH. This unexpected behaviour was also observed in the validation experiments [44].

As with the results shown in Fig 7, the position of the exhaust openings does not have a significant influence on *IF*. In 8 of the 12 cases analysed *IF* is clearly higher than *IF*_*PMV*_, the amount of inhaled contaminant, and therefore the risk of infection, is clearly higher than what would correspond to PMV. These results discourage the use of displacement ventilation in AIIRs.

### Comparison between indexes

Both *ε*_*a*_ and *ε*_*c*_ are global indices and provide valuable information for quantifying the quality of ventilation in the whole room, regardless of the ACH. However, the spatial distribution and temporal evolution of both the contaminant concentration and contaminant age are neither homogeneous nor static. In order to estimate the risk of infection, a local index such as the *IF* is more appropriate. The drawback, however, is that *IF* depends on ACH. If the aim is to compare cases with different ACH, it is necessary to normalise. One possible normalisation is with the *IF* that would correspond to a PMV, *IF*_*PMV*_. The three indices shown in Table 2 are independent of ACH.

**Table 2:** Indices.

Case	*ε*_*a*_	*ε*_*c*_	IFIF_PMV_
EN_12	0.56	1.74	2.24
ES_12	0.53	0.73	0.35
WN_12	0.70	1.65	2.52
WS_12	0.70	0.89	0.16
EN_09	0.59	1.14	0.72
ES_09	0.56	1.23	2.70
WN_09	0.71	1.29	0.14
WS_09	0.67	1.17	2.25
EN_06	0.56	1.61	1.89
ES_06	0.56	2.07	4.88
WN_06	0.68	1.43	2.00
WS_06	0.62	1.47	3.67

Table 2 shows that air change efficiency is not correlated either with contaminant removal effectiveness or intake fraction. There is also no clear correlation between contaminant removal effectiveness and intake fraction. The position of the HCW and the number of ACH have virtually no influence on air change efficiency The position of the exhaust openings has a greater impact on *ε*_*c*_ than on *ε*_*a*_, whereas changing the position of the HCW has a greater effect on *ε*_*a*_ than on *ε*_*c*_. Only in two of the twelve cases analysed is *IF/IF*_*PMV*_ clearly below 1. Therefore, although DV is a suitable strategy to renew the air in the room and to eliminate exhaled contaminants (high values of *ε*_*a*_ and *ε*_*c*_) it is not as effective at decreasing cross-infection risk.

## Conclusions

Detailed transient CFD tests have been carried to determine the suitability of a DV strategy in a representative case study of a one-bed hospital room. Two exhaust configurations, two external wall positions and three air renewal rates have been tested. In view of the results, the following conclusions may be drawn:

In most of the DV cases analysed in this paper, the values of air change efficiency and contaminant removal effectiveness are very promising. Nevertheless, analysing AIIR ventilation system performance based exclusively on global indices, such as air change efficiency or contaminant removal effectiveness, entails major limitations that can lead to erroneous decisions. Intake fraction provides more useful information in this type of rooms. This conclusion is supported by the lack of correlation between *IF* and the rest of the indices.

A priori, it could be assumed that an increase in the number of ACH leads to a decrease in the risk of infection. However, it has been found that an increase in the ventilation rate could not decrease exposure and in certain circumstances may indeed increase it. This conclusion concurs with other works [23,61,62]. The reason for this behaviour is unknown, and further analyses are required to understand the phenomenon. Local airflow pattern plays a crucial role in the transport and dispersion of exhaled pathogens and, consequently, the risk of cross-infection.

Numerous works have studied the impact of the position of the air inlets and exhaust openings or ACH in ventilation efficiency. In most of the works, the room envelope was considered to be well insulated and therefore adiabatic. This work has been shown that the presence of a radiant wall significantly affects the air flow pattern and contaminant dispersion. For the cases analysed, the relative position between the HCW and the radiant wall has a greater impact on the risk of infection than the extraction position or the number of ACH. When the radiant wall is opposite the HCW, the combined convective effect of the wall and the exhaled air drive the pollutant cloud away from the HCW.

In all the DV cases analysed, contaminants exhaled by the patient accumulate at the HCW inhalation height. This is the well-known lockup phenomenon. However, the air inhaled by the HCW comes from lower layers due to the effect of the convective boundary layer around the HCW. This argument has been used previously to recommend the use of DV in this type of room. However, despite this effect, the results obtained in this work do not advocate the use of DV. *IF* is no better than what may be expected for a PMV air flow pattern. It would be interesting to analyse the suitability of other ventilation strategies, such as downward-directed ventilation, for airborne infection isolation rooms.

## Limitations of the work

This work fails to consider either human movement or the movement of opening and closing doors. An analysis of how human movement affects the air flow pattern of displacement ventilation would prove extremely valuable. A further limitation is that the external wall is simulated with only one heat gain value. In a real situation, heat gain changes during the day and during the seasons. In addition, the study only analyses the exhalation of small respiratory bio-aerosols during breathing. Taking into account other pulmonary activities, such as coughing or sneezing, which expel larger droplets with higher initial momentum, would provide further insights.

Microbial survival in the environment is another issue not dealt with as it also lies beyond the scope of this article. The results obtained and discussed in this work are obtained under specific conditions which, whilst representative of the most common situations, do not represent every possible scenario. For instance, if the distance between the people or the height at which the contaminants are exhaled were changed then exposure to the contaminants might change. Despite these limitations, however, the results might prove helpful in the design of AIIRs in order to implement ventilation strategies where the exposure to exhaled contaminants may be reduced in most situations.

## Abbreviations

ACH: Air change per hour, h^-1^
AIIR: Airborne infection isolation room
B: Bed
CFD: Computational fluid dynamics
DD: Displacement diffuser
DV: Displacement ventilation
E: Exhaust openings located on the upper part of the east wall
EN: Ventilation system configuration combining E and N
ES: Ventilation system configuration combining E and S
HCW: Health care worker
IF: Intake fraction index
IF_PMV_: Intake fraction index with PMV
MV: Mixing ventilation
N: External wall heat gain on north wall
P: Patient
PMV: Perfect mixing ventilation
PDV: Perfect displacement ventilation
Q: Air flow rate in the room, m^3^/s
RANS: Reynolds-Averaged Navier-Stokes equations
S: External wall heat gain on south wall
SM: Source manikin (P)
TM: Target manikin (HCW)
URANS: Unsteady-RANS
W: Exhaust openings located on the upper part of the west wall
WN: Ventilation system configuration combining W and N
WS: Ventilation system configuration combining W and S
c: Average concentration of contaminant in the room
c_e_: Contaminant concentration in the exhaust air
D_a_: Mass diffusion coefficient, m^2^/s
ε_a_: Air change efficiency index
ε_c_: Contaminant removal effectiveness
*l*_0_: Largest scale of the room, m
$\bar{\tau }$: Local mean age of the air, s
τ_n_: Nominal time constant, s
V: Room volume, m^3^
*v*_0_: Characteristic air velocity, m/s
Y_P_: Mass fraction of N_2_O in the air exhaled by P
Y_PMV_: Mass fraction of N_2_O in a room with PMV
